# Relationship Between CNVs and Immune Cells Infiltration in Gastric Tumor Microenvironment

**DOI:** 10.3389/fgene.2022.869967

**Published:** 2022-06-08

**Authors:** Fazhan Li, Huijuan Wen, Ihtisham Bukhari, Bin Liu, Chenxu Guo, FeiFei Ren, Youcai Tang, Yang Mi, Pengyuan Zheng

**Affiliations:** ^1^ Henan Key Laboratory of Helicobacter Pylori, Microbiota and Gastrointestinal Cancer, Marshall Medical Research Center, The Fifth Affiliated Hospital of Zhengzhou University, Zhengzhou, China; ^2^ Academy of Medical Science, Zhengzhou University, Zhengzhou, China; ^3^ Department of Pediatrics, The Fifth Affiliated Hospital of Zhengzhou University, Zhengzhou, China; ^4^ Department of Gastroenterology, The Fifth Affiliated Hospital of Zhengzhou University, Zhengzhou, China

**Keywords:** gastric cancer, cancer immunotherapy, immune infiltration, copy number variation, tumor microenvironment

## Abstract

Gastric cancer (GC) is a highly fatal and common malignancy of the digestive system. Recent therapeutic advancements have significantly improved the clinical outcomes in GC, but due to the unavailability of suitable molecular targets, a large number of patients do not respond to the immune checkpoint inhibitors (ICI) therapy. To identify and validate potential therapeutic and prognostic targets of gastric cancer, we used the “inferCNV” R package for analyzing single-cell sequencing data (GSE112302) of GC and normal epithelial cells. First, by using LASSO, we screened genes that were highly correlated with copy number variations (CNVs). Therefrom, five gene signature (*CPVL*, *DDC*, *GRTP1*, *ONECUT2*, and *PRSS21*) was selected by cross-validating the prognosis and risk management with the GC RNA-seq data obtained from GEO and TCGA. Moreover, the correlation analyses between CNVs of these genes and immune cell infiltration in gastric cancer identified *CPVL* as a potential prognostic marker. Finally, *CPVL* showed high expression in gastric cancer samples and cell lines, then siRNA-mediated silencing of *CPVL* expression in gastric cancer cells showed significant proliferation arrest in MGC803 cells. Here, we conclude that CNVs are key regulators of the immune cells infiltration in gastric TME as well as cancer development, and *CPVL* could potentially be used as a prognostic and therapeutic marker in gastric cancer.

## Introduction

Gastric cancer is categorized as the fourth most common and third cause of cancer-related mortality worldwide (Bray et al., 2018). In recent years, the survival rate in many cancers has been improved due to the application of neoadjuvant chemotherapy, but in GC it is still worst because of its diagnosis at advanced stages, drug resistance, recurrence (Pernot et al., 2015; Ilson, 2017; Zurleni et al., 2018), high tumor heterogeneity, and poor immune cell infiltration in TME, which seriously hinder the prognosis and therapeutic outcomes (Meacham and Morrison, 2013; Weinberg, 2014; Van Cutsem et al., 2016). Therefore, it is crucial to thoroughly explore the heterogeneity of the GC, the mechanism of immune cell infiltration, and the new therapeutic targets for gastric cancer.

Currently, transcriptomic analyses revealed a variety of biomarkers that provide a base to determine the complexity and heterogeneity of tumors and identify new therapeutic targets (Cieslik and Chinnaiyan, 2018). The large-scale copy number variations (CNVs) through single-cell RNA-seq (scRNA-seq) have distinguished malignant cells from the normal cells (Patel et al., 2014; Müller et al., 2016; Tirosh et al., 2016). In general, the CNVs may regulate the function of somatic cells thus it is useful to use as the potential tumorigenesis markers (Beroukhim et al., 2010) and may affect immunotherapy (Kacew et al., 2019). On the other hand, the regulation of immune cell infiltration in TME plays an important role in the occurrence, progression, therapeutics and prognosis of cancers including GC (Siegel et al., 2017; Waniczek et al., 2017). However, the role of CNVs in immune cell infiltration and tumor development specifically in gastric cancer is not well illustrated.

Certainly, the scRNA-seq is an effective method for analyzing the heterogeneity of the complex biological systems such as TME (Chung et al., 2017; Cochain et al., 2018). Therefore, we quantified CNVs from scRNA-seq data of the gastric cancer and surrounding normal tissues to differentiate malignant cells from the normal epithelial cells. The differentially expressed genes (DEGs) in GC tissues were screened by the least absolute shrinkage and selection operator (LASSO) Cox regression model (McEligot et al., 2020). The expression of the hub genes and their CNVs in gastric cancer were further assessed for their relationship with immune cell infiltration and prognosis in the gastric tumor. Lastly, we determined the expression of levels of all hub genes in gastric cancer tissues and cell lines, then based on the expression pattern of the genes we selected CPVL to explore its role in cellular proliferation in gastric cancer cell lines.

## Methods and Materials

### Data Mining and Processing

**FIGURE 1:**
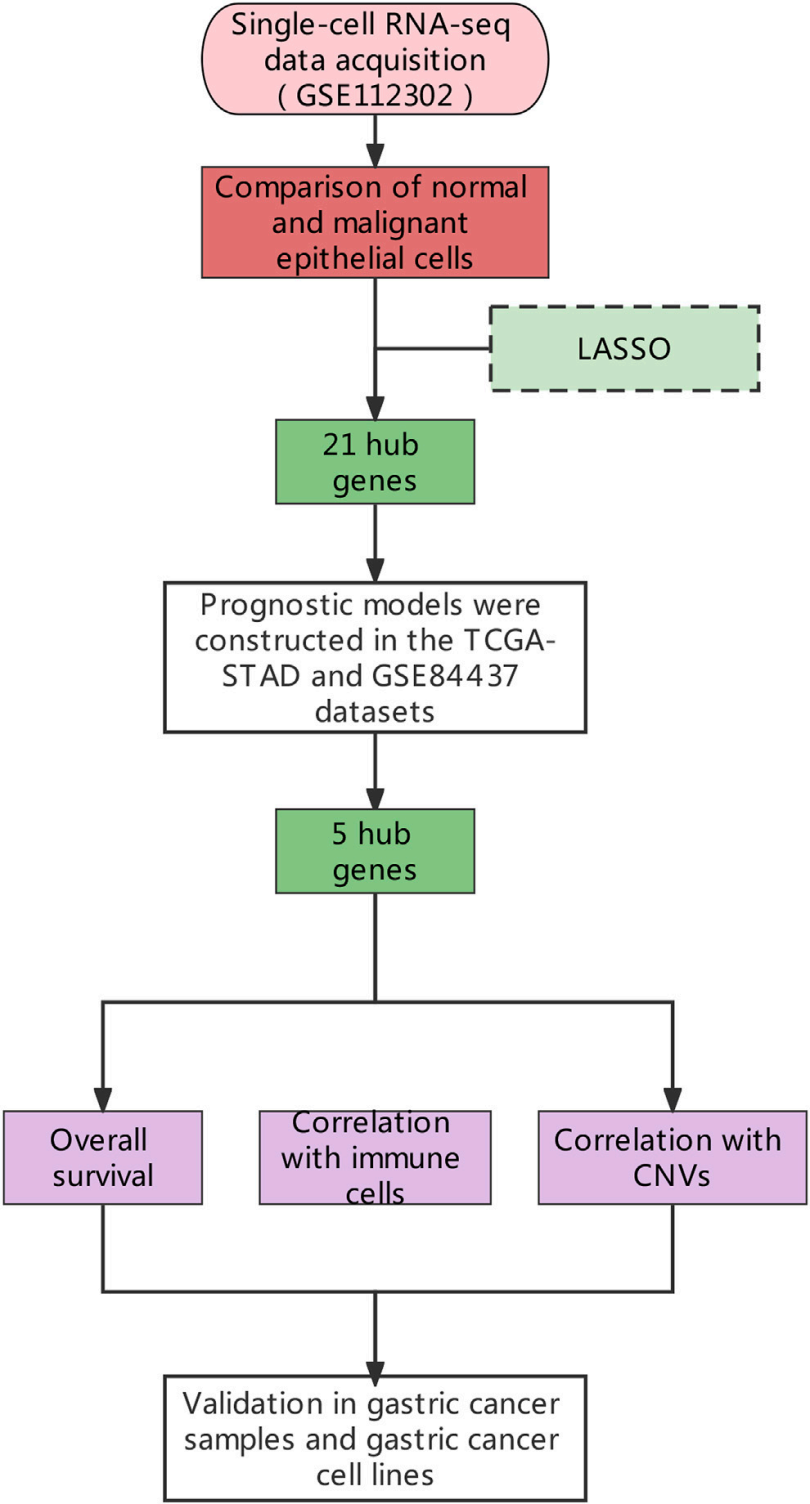
The general research design.

The scRNA-seq data of a total of 707 tumors and paired normal cells (GSE112302), and RNA-seq data (GSE84437) of 357 gastric cancer tissues were downloaded from Gene Expression Omnibus (GEO: https://www.ncbi.nlm.nih.gov/geo/) database. The transcriptome sequencing data of 407 individuals (32 normal and 375 gastric cancer tissue samples) along with complete clinical information were downloaded from The Cancer Genome Atlas (TCGA: https://portal.gdc.cancer.gov/). The complete study design and sample information are shown in Figure 1. The Seurat (Loraine et al., 2015) in RStudio (Stuart et al., 2019) was used to filter and standardize the tumor samples (355 tumor cells in total) in GSE112302 single-cell sequencing data. Genes expressed in less than three samples, and mitochondrial (MT) genes expressing >5% samples were filtered out. Principal component analysis (PCA) and tSNE were used for the dimensional reduction (Satija et al., 2015), and different cells’ clusters were identified and annotated according to the marker genes (resolution = 0.5). The raw data of GSE84437 and the gastric cancer data from TCGA were standardized using the “limma” package (Smyth et al., 2005).

### Epithelial Cell Reanalysis

The CNVs in macrophages (*n* = 23) and epithelium (*n* = 332) were evaluated by “infercnv” R package (Patel et al., 2014). The genomic locations of the differential genes with a high rate of copy number variations (CNVs) were determined and compared their average expression level with their relative expression. The “limma” package was used to find the differentially expressed genes among tumor epithelial cells with high and low CNVs (|log FC|> 2, *p* < 0.05).

### LASSO Regression Analysis

We constructed a prediction model based on the CNV scores of five signature genes by performing a LASSO regression analysis using the “glmnet” package in R. LASSO regression was applied to the module(s) with *p*-value of cnv-scores less than 0.05 to determine the signature gene, and the analyses were repeated 1,000 times to cross-validate and to select the best lambda value of the hub gene.

### Construction of the Prognostic Model

The risk scores of the potential genes from both TCGA transcriptome and GSE84437 data of gastric cancer were calculated by the formula Risk score = βgene1 × exprgene1 + βgene2 × exprgene2 + · ···· + βgene *n* × exprgene *n*. By considering the median risk score as a critical value, the samples were divided into high-risk groups and low-risk groups. Overall survival was determined by using R packages “survival” and “survminer” while R package “survival ROC” was used to draw the receiver operating characteristic curve (ROC), and Area Under Curve (AUC) to show the accuracy of the prognostic model. Single-factor COX analysis and multi-factor COX analysis were used to analyze the relationship between risk scores and clinical characteristics. R packages “rms” were used to build a nomogram model to estimate the factors related to overall survival and to calculate the risk scores. The sum of risk scores was used to predict 3-years and 5-years survival rates.

### Immune Relevance of Hub Genes

Kaplan-Meier plotter (http://kmplot.com/analysis/) is an online database for analyzing the survival rate of mRNA or miRNA in various cancers. Using this, we obtained the survival curve of the hub genes in gastric cancer, and their correlation with the infiltration of the immune cells (neutrophils, macrophages, dendritic cells, B cells, and CD4/CD8 T cells) in the tumor microenvironment was analyzed by TIMER (https://cistrome.shinyapps.io/timer/) (Li et al., 2017). The expression levels of the hub genes were also analyzed in the pan-cancer to find the difference between normal tissues and various cancers. The differences in the genes’ expression and the relationship of hub genes with immune cell infiltration and immune checkpoints (*PDCD1*, *CD274*, *PDCD1LG2*, and *CTLA4*) were also determined.

### Gastric Cancer Tissue Sampling, RNA Extraction and Q-RT-PCR Analyses

A total of seven gastric cancer tissue and adjacent normal tissue samples were obtained from patients undergoing partial gastrectomy at the Fifth Affiliated Hospital of Zhengzhou University. Informed written consent was obtained from all patients before recruitment into the study, and all sampling and experimental procedures were approved by the ethics committee of the fifth affiliated hospital of Zhengzhou University. Fresh tissues were washed thrice with PBS, crushed and treated with TRIzol (Invitrogen, Carlsbad, United States) for RNA extraction. The cDNA samples were prepared by using the Rever Tra Ace qPCR RT Kit (Osaka, Japan) and mRNA expression was performed using SYBR Premix Ex Taq (Tokyo, Japan) and specially designed primers (Table 1). GAPDH was used as internal control and the relative transcriptional expression level was calculated according to the 2^^∆^Ct approach.

**TABLE 1:** List of the primers used in the study.

Primer Name	Sequence
CPVL-F	TGA​CCT​TGC​GTG​ACA​GAG​AC
CPVL-R	CCG​TGC​ACC​GCA​AAA​AGT​TA
DDC-F	TGG​GGA​CCA​CAA​CAT​GCT​G
DDC-R	TCA​GGG​CAG​ATG​AAT​GCA​CTG
GRTP1-F	GGC​TAC​TAC​CAC​CAG​CTT​CTC​CAG
GRTP1-R	AAG​GTC​CGG​TTC​AGG​TCT​GTC​C
PRSS21-F	GCA​ACC​ACC​TCT​TCC​TCA​AGT​ACA​G
PRSS21-R	CTG​AGT​CAC​CGA​AGC​AGG​CAT​C
ONECUT2-F	TAC​TGC​CAC​ACC​CAT​ACG​AGA​GAG
ONECUT2-R	TGC​TGA​GGA​GAG​GTC​TGC​CAA​G
GAPDH-F	GAG​TCA​ACG​GAT​TTG​GTC​GT
GAPDH-R	TTG​ATT​TTG​GAG​GGA​TCT​CG
*CPVL*-SiRNA1- sense	5′-CGC​UCU​CCA​UGC​UUU​ACA​UTT-3′
*CPVL*-SiRNA1- antisense	5′-AUG​UAA​AGC​AUG​GAG​AGC​GTT -3′
*CPVL*-SiRNA2- sense	5′-CAG​CUU​UAC​UAU​GUG​AAA​UTT-3′
*CPVL*-SiRNA2- antisense	5′-AUU​UCA​CAU​AGU​AAA​GCU​GTT-3′
hsa-miR-196b-5p	GTC​GCA​TCA​AGG​ATC​TTA​AAC​TTT​GCC
hsa-miR-561-5p	ACG​CTA​GGT​AGT​TTC​CTG​TTG​TTG​G
hsa-miR-7-5p	ACG​CGT​GGA​AGA​CTA​GTG​ATT​TTG​TTG
hsa-miR-196a-5p	ACG​CTA​GGT​AGT​TTC​ATG​TTG​TTG​GG
LINC01678-F	CCC​TCC​TGC​AAC​AAT​CGG​G
LINC01678-R	GGA​CCA​GGG​TAG​TGA​GGT​AGT
TRG-AS1-F	GCC​TGA​ATC​CAG​TGT​TCC​TGA​GTG
TRG-AS1-R	TGG​GCA​TTG​GTC​TTG​GCT​TTG​TC
LINC00472-F	TTC​AGG​AAT​ATG​GCA​GGC​TCA​ACA​C
LINC00472-R	GGG​TGG​TCT​GAG​AGG​AGG​CAT​C
AL158207.2-F	ACC​ACT​CAT​CAA​ATC​CTG​CCA
AL158207.2-R	GGA​GCA​CTT​AGC​ACC​GCA​TA
AL122035.2-F	CAG​GAA​TAT​GGC​AGG​CTC​AA
AL122035.2-R	GGG​CAT​TGG​TCT​TGG​CTT​TGT

### Correlation of *CPVL* With Immune and Matrix Scores

The “Estimate” package was used to evaluate the immune score and matrix score in pan-cancer. The correlation between *CPVL*, immune, and matrix scores was calculated by spearman text and the “ggplot2” package was used to draw scatter plots.

### siRNA-Mediated Gene Knockdown

The siRNA targeting *CPVL* and scrambled negative control siRNA were provided by Shanghai GenePharma (Shanghai, China), the sequences of siRNA are listed in Table 1. The MGC803 cells were transfected with siRNA using Lipofectamine 2000 (Invitrogen, Carlsbad, CA, United States) at a final concentration of 80 nM. The efficiency of siRNA knockdown was subsequently confirmed using qPCR.

### Cell Proliferation Assay

Cell Counting Kit-8 (DOJINDO, Kumamoto, Japan) was used according to the manufacturer’s instructions. After 24–48 h of transfecting cells with siRNA-CPVL, ∼5,000 cells were seeded into each well of the 96-well plate, followed by adding 10 μL of CCK-8 reagent to each well after 24 h and incubating at 37°C for 2 h, then measuring absorbance at 450 nm (ELx800, Bio-Tek, United States). Starting from siRNA transfection to the proliferation test, all experiments were repeated thrice.

### The Prediction of *CPVL* Related miRNA and Upstream lncRNA

The StarBase (http://starbase.sysu.edu.cn/) is an online database for studying correlation and predicting the regulatory relationships between mRNA, miRNA, and lncRNA in tumors (Li et al., 2014). First, we predicted the candidate miRNAs associated with *CPVL* by taking |R|> 0.1 and *p*-value <0.05 as a baseline, however, miRNAs showing higher significance were chosen and validated by qPCR in gastric cancer tissues and cell lines.

### Statistical Analysis

R package “Seurat” and “infercnv” were used to perform quality control on single-cell sequencing data, and to filter and calculate cell cnv-scores. The expression levels of genes in transcriptome data were normalized by log2 transformation. COX regression analysis was used to establish the relationship between gene expression and risk score, R package “survival ROC” for survival prediction models, and the “rms” package was used to build nomogram models. The “Estimate” package was used to evaluate the immune score and matrix score of pan-cancer, and the Spearman test was used to calculate the correlation between *CPVL* and the immune score and matrix score. *p* < 0.05 was considered as significant.

The experimental data were statistically analyzed and plotted by GraphPad 8.0 software, and other measurement data were described by means ± standard deviation, all using the student t-test.

## Results

### CNV and Related Genes Within the Tumor Cell Through scRNA-Seq Analyses

**FIGURE 2:**
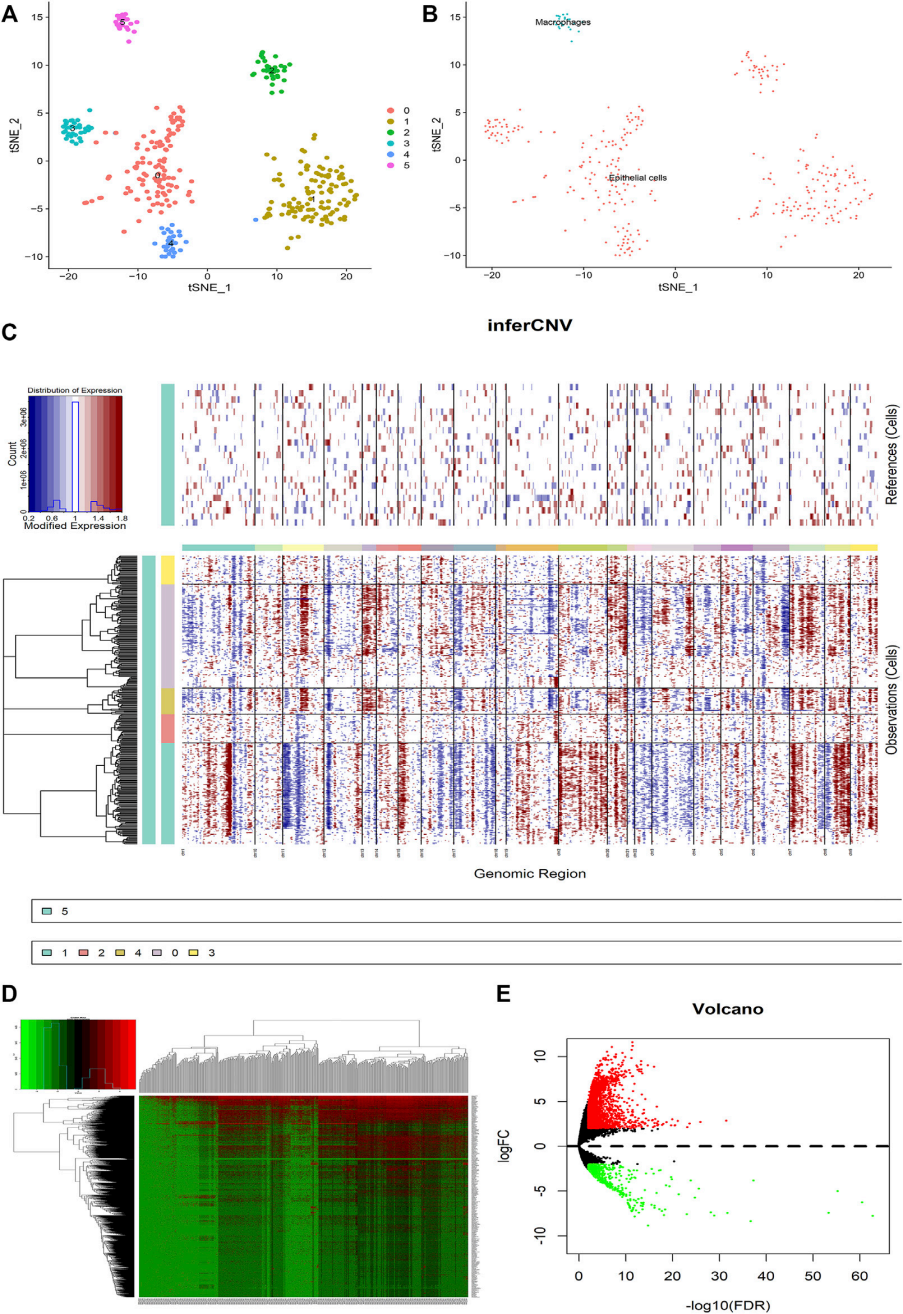
Analysis of the difference between malignant cells and normal epithelial cells in GC tumors. **(A)** The tumor cells are clustered into six clusters including 0–5, clusters 0–4 are annotated as epithelial cells, and cluster 5 is annotated as macrophages. **(B)** The cells are divided into epithelial cells and macrophages. **(C)** CNV heat map of other epithelial cells with cluster 5 macrophages as a control. **(D,E)** Heat map and volcano map for differential analysis. Red represents upregulation and green downregulation, |logFC|>2, *p* < 0.05.

First of all, the quality checks were performed for the scRNA-seq GSE112302 dataset (Supplementary Figures S1A,C), and selected the top 1,500 genes fulfilling our basic selection criteria (Supplementary Figure S1B). For 355 tumor cells, we performed principal component analysis (PCA) and clustered the cells using graph-based clustering (*n* = 20) (Supplementary Figures S1D–G). Furthermore, we annotated the cells including epithelial cells and macrophages showing marker genes (Figures 2A,B). It has been known that the transformation of normal cells to cancer cells is closely related to large-scale chromosomal aberrations (Beroukhim et al., 2010). Therefore, the CNVs in different genes were analyzed to differentiate between normal epithelial cells and cancer cells, and we found that cluster2 and cluster3 have comparatively low CNVs than other clusters 0, 1 and 4 (Figure 2C). Furthermore, we analyzed the differences between the tumor cells expressing high and low CNVs, showing a large number of differential genes in both groups (Figures 2D,E). These results indicate that CNVs are one of the key contributors to GC heterogeneity.

### LASSO Regression Analysis

**FIGURE 3:**
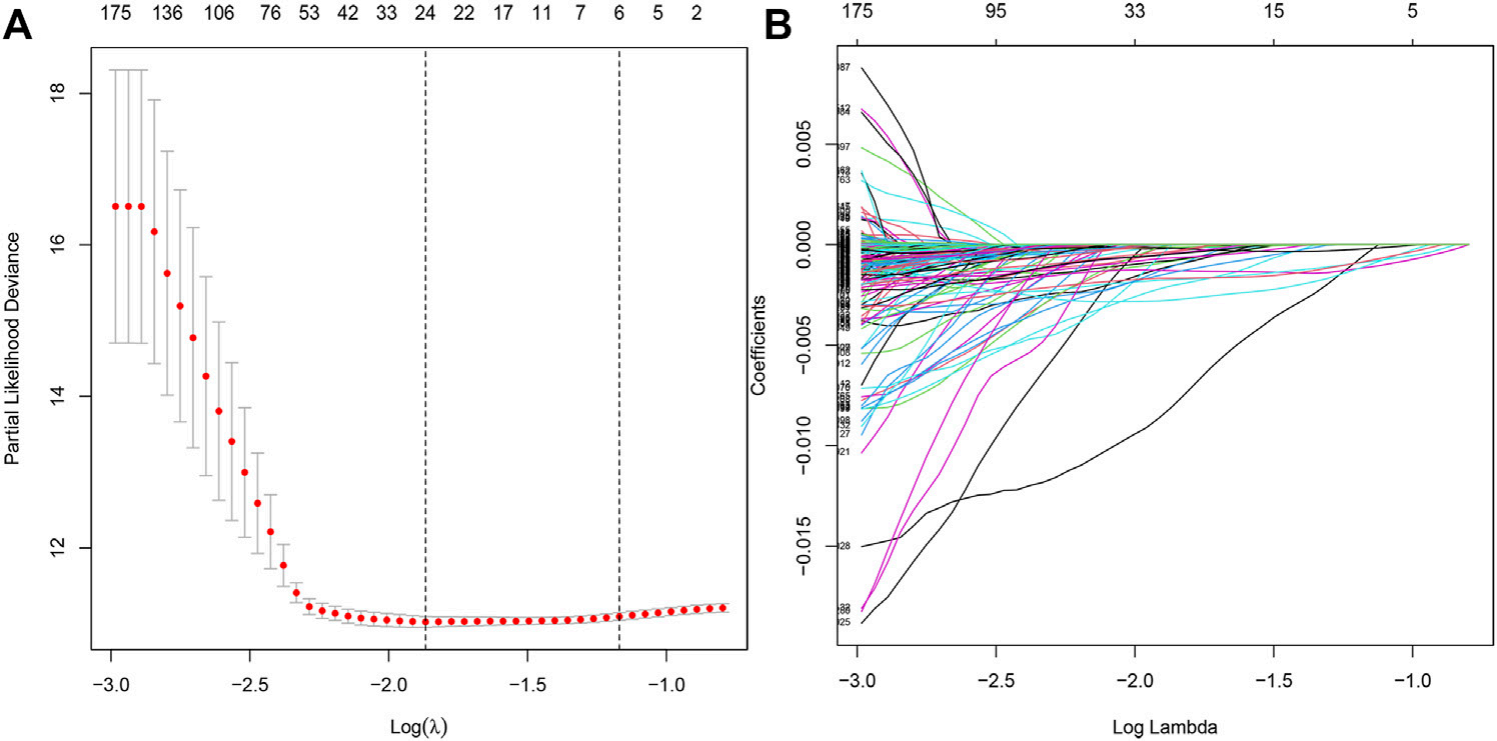
LASSO regression analysis model construction. **(A)** LASSO coefficient distribution of differentially expressed genes. **(B)** Partial likelihood bias of the LASSO coefficient distribution.

Furthermore, we performed the LASSO regression analysis for the differential genes (Figures 3A,B), and obtained 21 genes as potential hub genes (*LRMP*, *SDR42E1*, *ERICH5*, *NPPC*, *KCNJ3*, *CNTNAP2*, *LINC00346*, *CPVL*, *UNC93A*, *ONECUT2*, *MAGEA6*, *SLC19A3*, *DDC*, *VGF*, *LINC00392*, *AQP2*, *PRSS21*, *ETV4, F12*, *RAP2A*, and *GRTP1*), suggesting a strong functional association of these genes with tumor epithelial cells.

### Hub Genes Correlated With CNV and Prognosis

**FIGURE 4:**
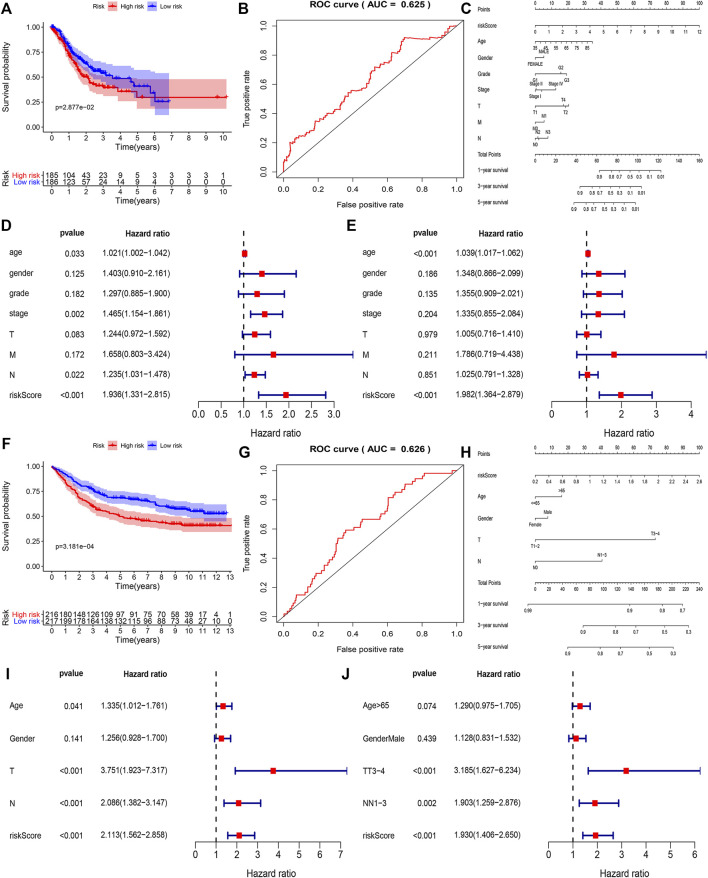
The correlation between the construction of the prognostic model and the clinical characteristics of the selected genes in the TCGA and GEO databases. **(A,B)** Based on COX regression analysis to screen out high-risk genes in TCGA, ROC curve showing accuracy (AUC = 0.625) of the clinical prognosis model. **(C)** Nomogram of TCGA risk score and clinical characteristics of gastric cancer patients. Each factor corresponds to its score, and each score is added to obtain a total score. **(D,E)** Single-factor COX regression analysis and multi-factor COX regression analysis of TCGA risk score. **(F,G)** Based on COX regression analysis a clinical prognostic model was constructed, and the ROC curve is used to detect the accuracy of the model (AUC = 0.625). **(H)** Nomogram of GSE84437 risk score and clinical characteristics of patients with gastric cancer. **(I,J)** Univariate Cox regression analysis and multivariate COX regression analysis of GEO risk score.

To find out the role of hub genes in the overall survival of gastric cancer we performed a multivariate Cox regression analysis, among 21 hub genes, the eight genes (*KCNJ3*, *CPVL*, *ONECUT2*, *SLC19A3*, *DDC*, *PRSS21*, *F12*, and *GRTP1*) were designated as independent indicators of poor prognosis. Based on these findings we constructed a prediction model for the clinical prognosis of GC patients and validated it by the ROC curve [0.625 as an area under the curve (AUC)] (Figures 4A,B). Next, the survival information of hub genes and clinical features of the patients were combined to construct the nomogram (Figure 4C). The total score of each prognostic factor was represented as 1, 3, and 5-years survival rates of the patients. To find out whether the risk signatures were independent predictors of prognosis, the Cox univariate analysis was performed showing that the age (*p* = 0.033), stage (*p* = 0.002), N stage (*p* = 0.022), and risk score (*p* < 0.001) were significantly linked with OS. While in Cox multivariate analysis only age (*p* < 0.001) and risk score (*p* < 0.001) were significantly linked with OS (Figures 4D,E).

**FIGURE 5:**
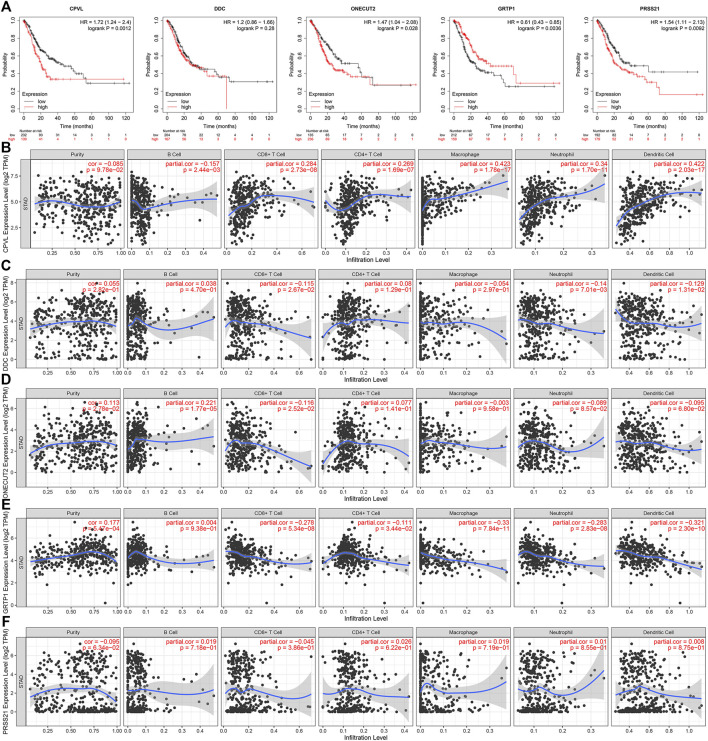
The survival curve of the Hub gene and its relationship with immune cell infiltration. **(A)** The survival curve of *CPVL*, *ONECUT2*, *DDC*, *PRSS21*, and *GRTP1* in patients with gastric cancer. **(B–F)** The correlation of *CPVL*, *ONECUT2*, *DDC*, *PRSS21*, and *GRTP1* with neutrophils, macrophages, dendritic cells, B cells, and CD4/CD8 T cells in gastric cancer immune infiltration.

Using the GSE84437 dataset, we identified five genes (*CPVL*, *ONECUT2*, *DDC*, *PRSS21*, and *GRTP1*) as independent indicators of poor prognosis. Based on that we constructed and validated a prediction model for clinical prognosis, ROC curve (AUC = 0.626) (Figures 4F,G), and the relationship between multiple factors and survival rate was analyzed by nomogram (Figure 4H). The single factor analysis in GSE84437 showed that age (*p* = 0.041), T stage (*p* < 0.001), N stage (*p* < 0.001), and risk score (*p* < 0.001) were significantly associated with OS. However, the Cox multivariate analysis showed a significant association between the T3-4 stage (*p* < 0.001), N1-3 stage (*p* = 0.002), and risk score (*p* < 0.001) with OS (Figures 4I,J). A venn diagram was drawn for these five survival-related genes (Supplementary Figure S2A), expressed in all cell clusters (Supplementary Figure S2B). Next, we plotted the relationship between the top five hub genes and survival in gastric cancer on the Kaplan-Meier plotter (http://kmplot.com/analysis/) (Figure 5A) showing a significant correlation between low expression of *CPVL*, *ONECUT2*, and *PRSS21* with prolonged survival of the patients. On the other hand, the low expression of *GRTP1* showed an association with poor prognosis, and the expression of DDC did not affect the prognosis of GC patients.

### Correlation Between Hub Genes and Immune Cells Infiltration

The copy number variations are one of the well-known factors affecting the infiltration of immune cells in tumor microenvironment (Campos-Carrillo et al., 2020). Therefore, we studied the correlation between five hub genes and the infiltration of immune cells such as neutrophils, macrophages, dendritic cells, B cells and CD4/CD8 T cells in GC TME. Among all genes, *CPVL* and *GRTP1* showed a significant positive and negative correlation with immune cell infiltration, respectively. Other genes did not affect the infiltration of immune cells in GC TME (Figures 5B–F).

*PD1/PD-L1* and *CTLA-4* are important immune checkpoints for tumor immune escape, playing significant roles in tumor immunotherapy (Guo et al., 2021). Among hub genes, only *CPVL* was found to have a positive and *ONECUT2* negative correlation with the immune checkpoints (Supplementary Figure S3).

### The Relationship Between Hub Genes, Copy Number Variation and Immune Cell Infiltration

**FIGURE 6:**
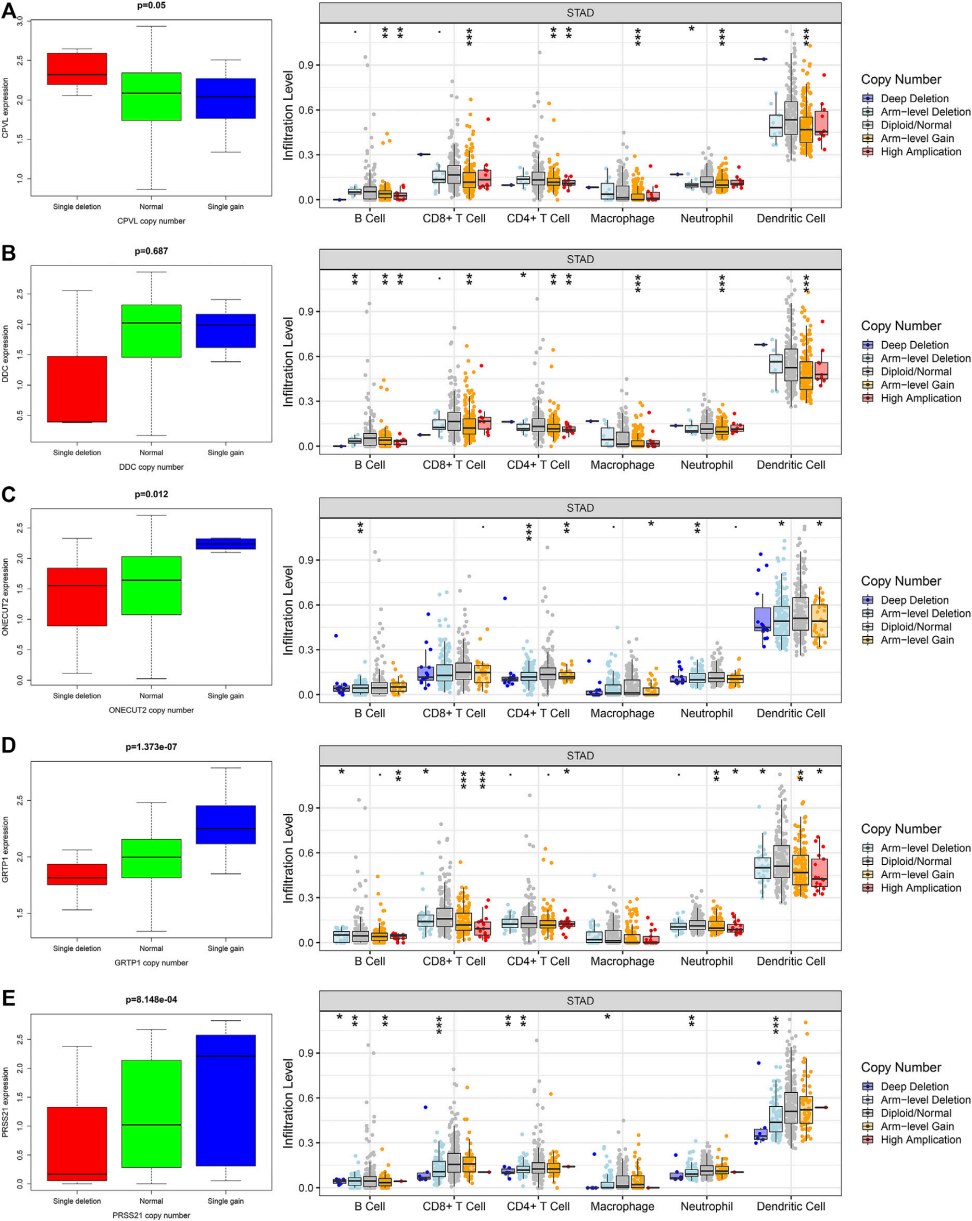
The relationship between the hub genes, CNVs and level of immune infiltration in gastric cancer. The expression and CNVs of **(A)** CPVL, **(B)** ONECUT2, **(C)** DDC, **(D)** PRSS21 and **(E)** GRTP1 showed association with the immune cell infiltration in gastric cancer. *p <0.05, **p <0.01, ***p <0.001.

Next, we explored the correlation between the expression of the genes and their CNVs with immune cell infiltration in gastric TME. For this purpose, we analyzed the somatic mutations of the hub genes through TIMER [deep deletion (-2), arm-level deletion (-1), diploid/normal (0), arm-level gain (1), and high amplification (2)] and the infiltration of the multiple immune cells. Interestingly, we observed that the copy number variations (CNVs) in hub genes significantly affect the infiltration of immune cells (Figures 6A–E). Apart from *DDC*, all other genes including *CPVL*, *ONECUT2*, *PRSS21*, and *GRTP1* positively regulate the infiltration of the immune cells. These findings revealed that CNVs are also major players in the immune regulation of the gastric cancer TME.

### Effect of CPVL on Proliferation of Gastric Cancer Cells

**FIGURE 7:**
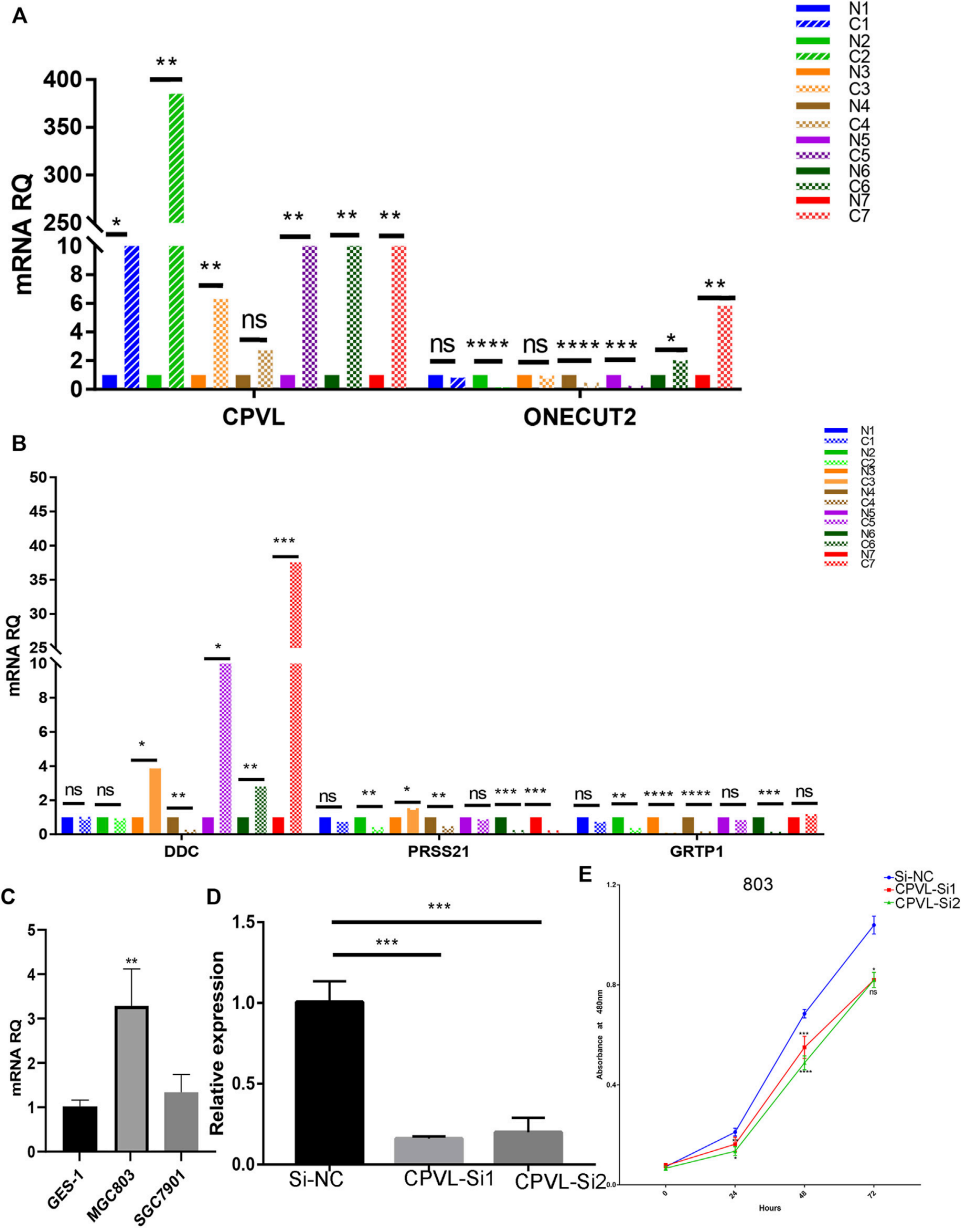
The effect of CPVL on cellular proliferation of Gastric cancer. **(A,B)** Q-PCR analysis of the relative expression levels of *CPVL*, *ONECUT2*, *DDC*, *PRSS21*, and *GRTP1* in gastric cancer and corresponding adjacent tissues. Where “C” represents the tumor samples and “N” represents the corresponding paracancerous samples. **(C)** Relative expression of CPVL in gastric cancer cell lines. **(D)** Knockdown efficiency of CPVL in MGC803. **(E)** Effects of CPVL knockdown on MGC803 proliferation. (*p < 0.05, **p < 0.01, ***p < 0.001, ****p < 0.0001).

We performed the expression of five hub genes in gastric cancers and normal tissue samples which showed significantly high expression of *CPVL* in cancer samples, and cell lines (Figures 7A–C) which was consistent with the RNA sequences obtained from TCGA. To further explore the role of *CPVL* in gastric cancer, two different siRNAs were used to silence the expression of *CPVL* in MGC803 cells (Figure 7D). Subsequently, we examined the effect of the *CPVL*-knockdown on the proliferation of MGC803 cells. The results showed a significant reduction in the proliferation of MGC803 cells after silencing *CPVL* (Figure 7E).

### Relationship of *CPVL* With Immunity in Pan-Cancer

**FIGURE 8:**
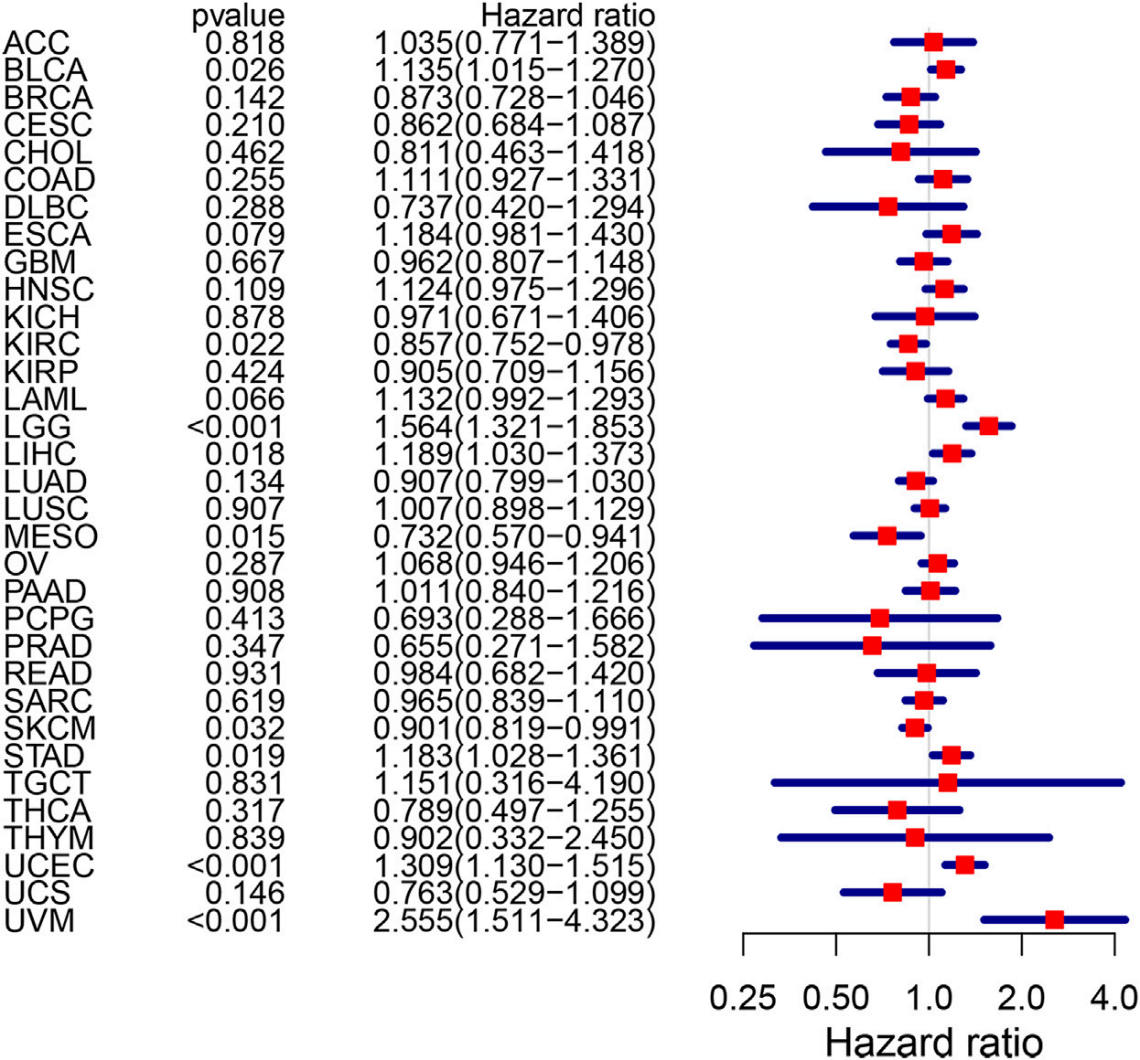
The risk rate of *CPVL* gene in pan-cancer. CPVL is associated with patients’ survival in BLCA, KIRC, LCC, LIHC, MESO, SKCM, STAD, UCEC, and UVM (*p* < 0.05). The hazard ratio greater than 1 indicates a high-risk gene(s), meaning that patients with high expression of such gene(s) have a worse prognosis; the hazard ratio less than 1 indicates a low-risk gene(s) for particular cancer, meaning that patients with high expression have a better prognosis.

Among five hub genes, we focused on *CPVL* because it showed the highest degree of correlation with immune cell infiltration and immune checkpoints *PD-1/PD-L1* and *CTLA-4* in gastric cancer. Thus, we comprehensively explored the *CPVL* in pan-cancer, and its high expression showed a strong association with the poor prognosis in a variety of cancers (Figure 8). Total out of 33 pan-cancers, *CPVL* was found to have high expression in 29 cancers (Supplementary Figure S4), also showing a significant correlation with the immune and matrix scores. These findings show that *CPVL* has a universal effect on the immune cell infiltration and immune function in TME of various cancers.

### The Prediction of *CPVL* Related miRNA and Upstream lncRNA

In starBase (http://starbase.sysu.edu.cn/), we have obtained four potential upstream miRNAs (*hsa-miR-196a-5p*, *hsa-miR-7-5p*, *hsa-miR-196b-5p*, and *hsa-miR-561-5p*) correlated with *CPVL* (Table 2) and among them, we selected *hsa-miR-196b-5p* as a potential target because of its highest degree of negative correlation with *CPVL*. Moreover, we predicted the upstream lncRNAs of *hsa-miR-196b-5p*, and obtained 68 possible lncRNAs, and five lncRNAs showing a negative correlation with *hsa-miR-196b-5p* and positive correlation with *CPVL* in gastric cancer were filtered out (Table 3). Furthermore, the expression levels of selected miRNAs and lncRNAs were determined in gastric cancer cell lines. The results showed that lncRNAs AL158207.2 and AL122035.2 were significantly reduced in gastric cancer samples, while the expression levels of the four miRNAs in gastric cancer cell lines were significantly higher than the normal controls (Supplementary Figure S5). Thus, based on these results, we believe that these lncRNAs and miRNAs could be used to construct ceRNA networks and studying other functions.

**TABLE 2:** Correlation between *CPVL* and miRNA.

geneName	Coefficient-R	*p*-value	
*hsa-miR-196a-5p*	*CPVL*	−0.09	8.33E-02
*hsa-miR-7-5p*	*CPVL*	−0.131	1.15E-02
*hsa-miR-196b-5p*	*CPVL*	−0.185	3.43E-04
*hsa-miR-561-5p*	*CPVL*	−0.088	8.97E-02

**TABLE 3:** Correlation analysis between lncRNA and hsa-miR-196b-5p or lncRNA and *CPVL* in GC determined by starBase database.

geneName	miRNAname	R	*p*-value	mRNA	R	*p*-value
*LINC00472*	hsa-miR-196b-5p	−0.381	2.71E-14	*CPVL*	0.166	1.24E-03
*TRG-AS1*	hsa-miR-196b-5p	−0.304	2.03E-09	*CPVL*	0.286	1.72E-08
*LINC01678*	hsa-miR-196b-5p	−0.28	4.03E-08	*CPVL*	0.124	1.59E-02
*AL158207.2*	hsa-miR-196b-5p	−0.226	1.07E-05	*CPVL*	0.147	4.40E-03
*AL122035.2*	hsa-miR-196b-5p	−0.205	6.79E-05	*CPVL*	0.082	1.15E-01

## Discussion

Gastric cancer is considered as one of the most deadly cancers, ranked as the third leading cause of cancer-associated deaths worldwide (Chen et al., 2014). Exploring the heterogeneity within the tumor can help to deeply understand the microenvironment of gastric cancer (Yan et al., 2018). Recently, it has been validated that CNVs (deletion, insertion or SNPs) in genes are one of the important factors affecting cellular functions (Nakamura, 2009). In a variety of tumors, including gastric cancer, a large number of CNVs are potentially involved in the occurrence and development of tumors (Leary et al., 2008; Despierre et al., 2014; Horpaopan et al., 2015; Xu et al., 2015).

A study using 183 gastric cancer samples identified drugs’ targeted genes exhibit a high ratio of copy number gains (CNG) (Labots et al., 2014). It is well-known fact that the CNVs play integral roles in the screening of new prognostic targets and individualized treatment for patients based on the heterogeneity of their tumors (Hou et al., 2016). Believing that, single-cell sequencing of tumors could serve as a useful tool for exploring tumor heterogeneity (Hou et al., 2016). In the current study, we considered the CNV-score of the cells to differentiate the malignant cells from epithelial cells in GC. Abnormally high expressing genes with CNV-score in the tumor cells were screened out, and top genes were analyzed for their roles in overall survival in gastric cancer patients. Furthermore, we also verified that the level of CNVs and high-risk scores related to the poor prognosis of gastric cancer patients.

The tumor immune process and the host’s immune protection mechanism are divided into three phases: elimination, equilibrium, and escape phases (Lazar et al., 2018). Specifically, at the early stages of tumorigenesis and development, the immune system can kill tumor cells to inhibit the development and progression of cancers. With the progression of the tumor, the surviving cancer cells become smarter and can change some of their characteristics to avoid killers’ immune cells thus proliferating and metastasizing. We found that high expression of *CPVL*, *ONECUT2*, *DDC*, *PRSS21*, and *GRTP1* increase the risk of gastric cancer. Interestingly, the expression of *CPVL*, *ONECUT2*, *PRSS21*, and *GRTP1* was linked with the higher ratio of CNVs which further suppressed the level of immune cell infiltration. These results partially explain that the low degree of immune cell infiltration in GC may affect the efficiency of ICIs. High expression of ONECUT2 and PRSS21 have been reported to be associated with poorer prognosis in various cancers including prostate cancer, hepatocellular carcinoma, and gastric cancer (Liu et al., 2021; Pollan et al., 2021; Shen et al., 2021; Sun et al., 2021).

In the current study, *CPVL* showed higher expression than the other genes, thus we selected it for further analyses. *CPVL* is a serine carboxypeptidase that was first characterized in human macrophages (Mahoney et al., 2001). Though, the function of *CPVL* remains unclear in a variety of tumors. So far *CPVL* has not been deeply studied in gastric cancer. However, *CPVL* induced apoptosis in glioma cells through the IFN-γ/STAT1 signaling pathway (Yang et al., 2021). In gastric cancer cell lines, we showed for the first time that *CPVL* on cell proliferation, suggesting that CPVL may be a new prognostic target for gastric cancer. Further comprehensive studies are required to explore its deep function and underlying mechanisms in regulation of gstric cancer.

## Conclusion

The high risk-scores of *CPVL*, *ONECUT2*, *DDC*, *PRSS21*, and *GRTP1* could be used to determine the degree of malignancy and prognosis in gastric cancer patients. The increased expression of *CPVL*, *ONECUT2*, *PRSS21*, and *GRTP1* may indicate the advanced stage of GC, as well as a low level of immune cell infiltration in gastric TME. In gastric cancer cell lines, we determined that *CPVL* regulate the cellular proliferation, showing that *CPVL* could be an important predictor in gastric cancer development.

## Data Availability

The datasets presented in this study can be found in online repositories. The names of the repository/repositories and accession number(s) can be found in the article/Supplementary Material.

## Supplementary Material

Supplementary Figure S1 Quality control and screening of single cells of gastric cancer. **(A)** Filter and normalize the tumor samples (355 tumor cells in total) in the GSE112302 single-cell sequencing data. **(B)** Red indicates the first 1,500 differential genes. **(C)** Sequencing depth and mitochondrial gene distribution. **(D–G)** Principal component analysis to screen marker genes (20 PCs in total).

Supplementary Figure S2 Take the intersection of the Venn diagram to obtain the hub gene and its expression in a single cell population.

Supplementary Figure S3 The scatter plot shows the correlation between *CPVL*, *ONECUT2*, *DDC*, *PRSS21*, and *GRTP1* genes and immune checkpoints (*PDCD1*, *CD274*, *PDCD1LG2*, and *CTLA4*) in gastric cancer.

Supplementary Figure S4 The scatter plot shows the correlation of *CPVL* with immune score and matrix score in pan-cancer.

Supplementary Figure S5 Relative expression levels of lncRNAs and miRNAs in gastric cancer cell lines.

## Abbreviations

AUC, Area under the curve; CNVs, copy number variations; DEGs, Differentially expressed genes; EBV+, Epstein-Barr virus infection positive; GEO, Gene Expression Omnibus; ICI, Immune checkpoint inhibitor; LASSO, Least Absolute Shrinkage and Selection Operator; MSI, Microsatellite Instability; PCA, Principal component analysis; ROC, Receiver operating characteristic curve; TCGA, The cancer genome atlas project; TME, Tumor microenvironment.
