# Supplementary figures and images for: Relationship Between CNVs and Immune Cells Infiltration in Gastric Tumor Microenvironment
Source: Front Genet. 2022 Jun 8;13:869967. doi: 10.3389/fgene.2022.869967 (PMC9214698; doi:10.3389/fgene.2022.869967)

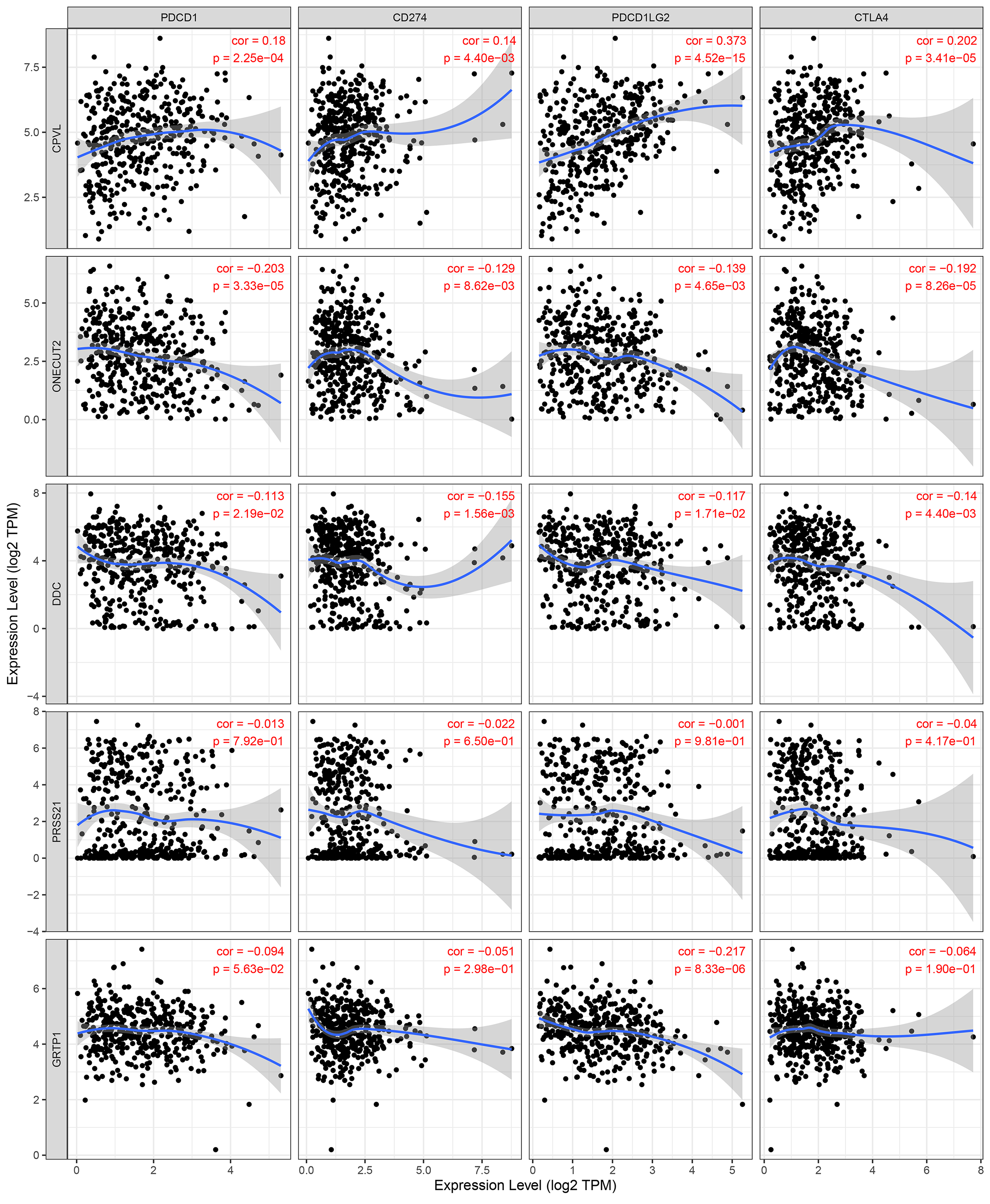

Supplement: Supplementary file 1 [file Image3.TIF]

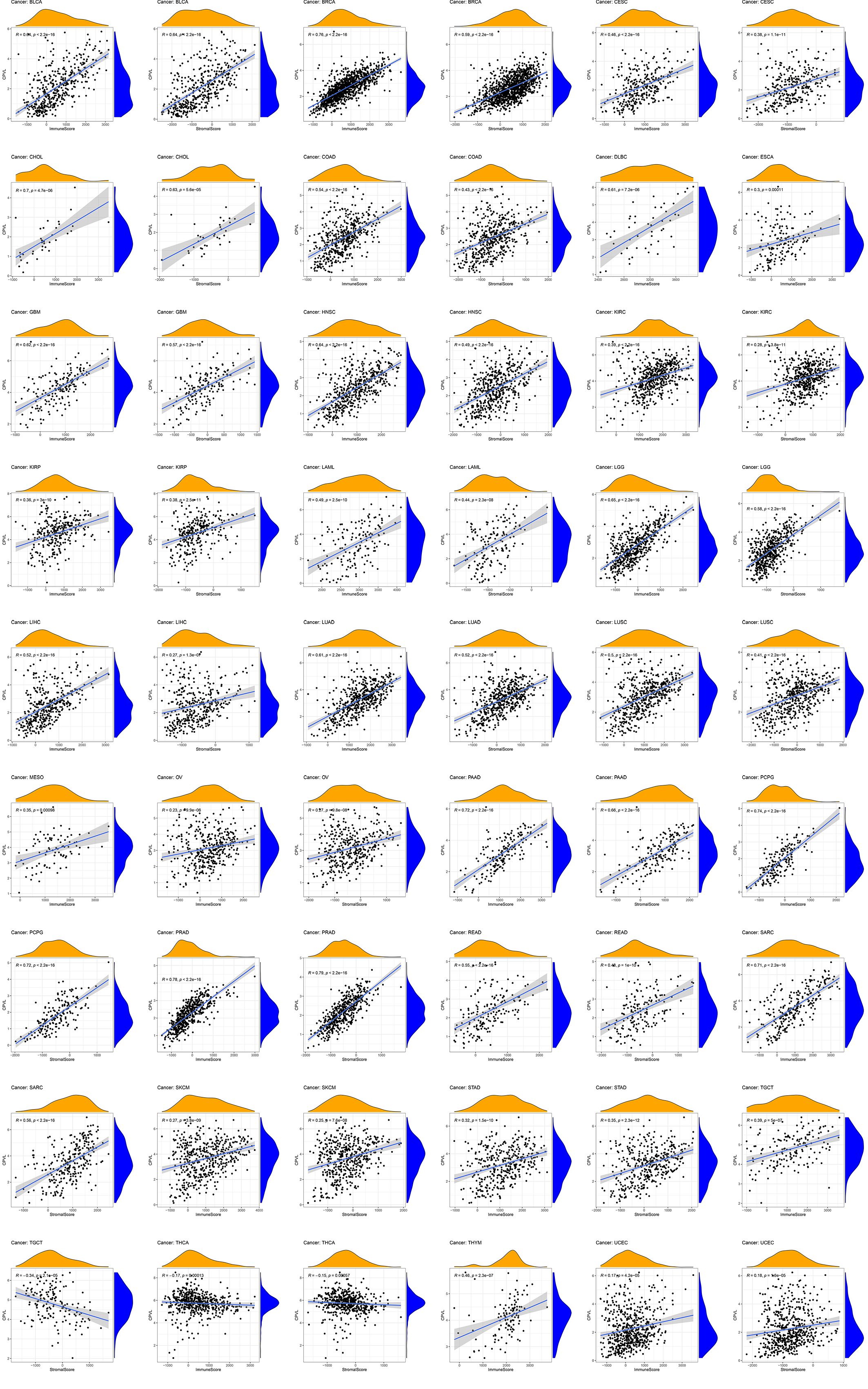

Supplement: Supplementary file 2 [file Image4.TIF]

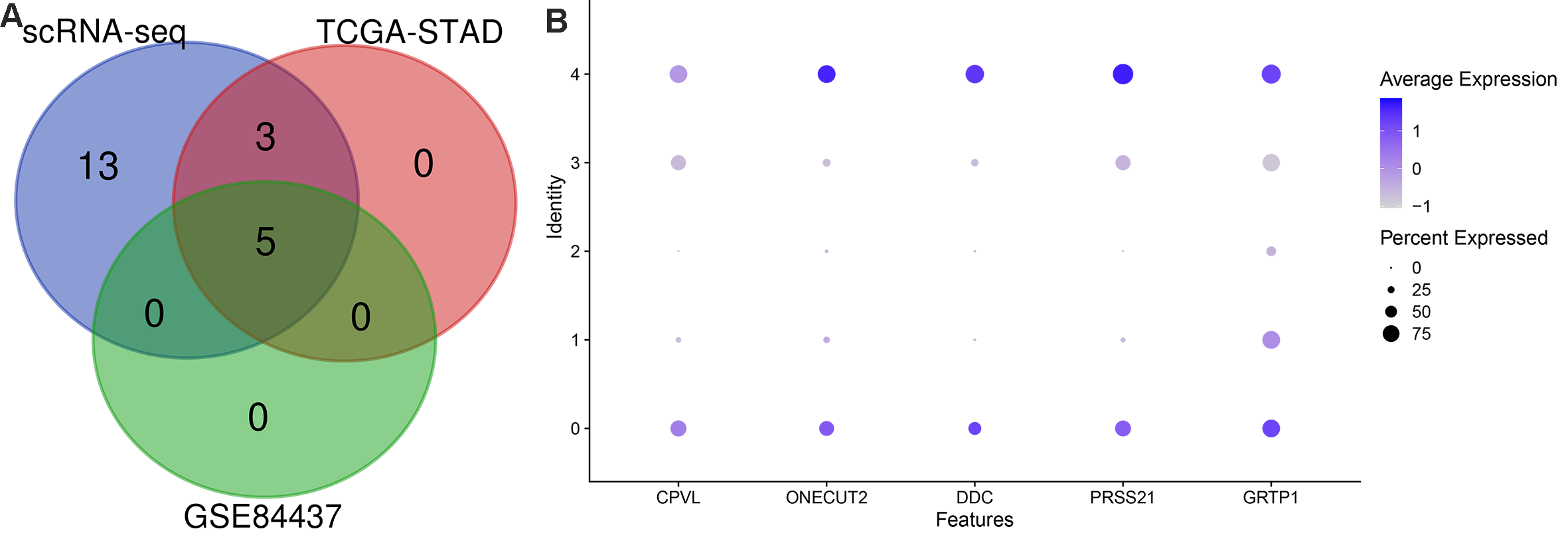

Supplement: Supplementary file 3 [file Image2.TIF]

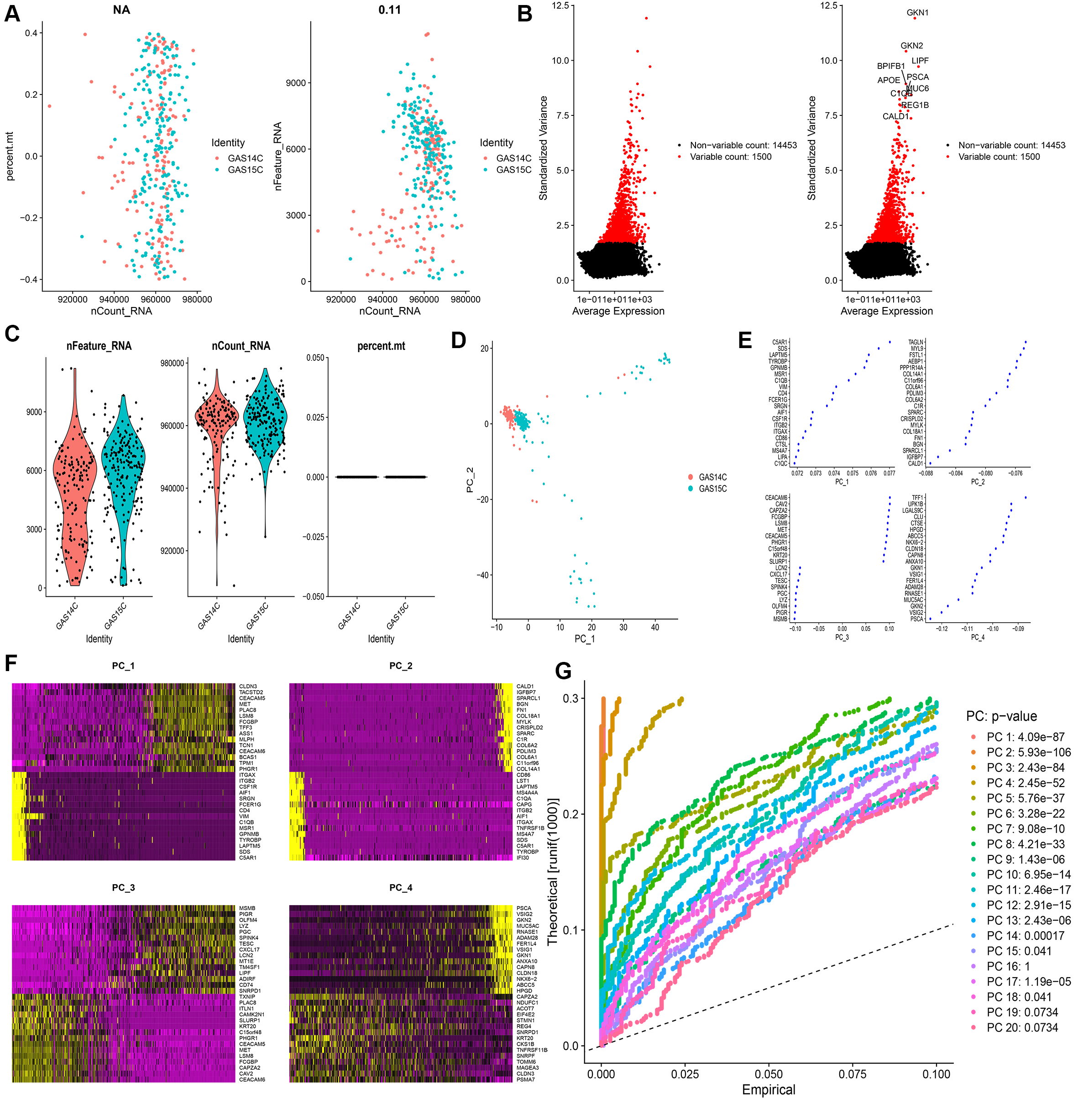

Supplement: Supplementary file 4 [file Image1.TIF]

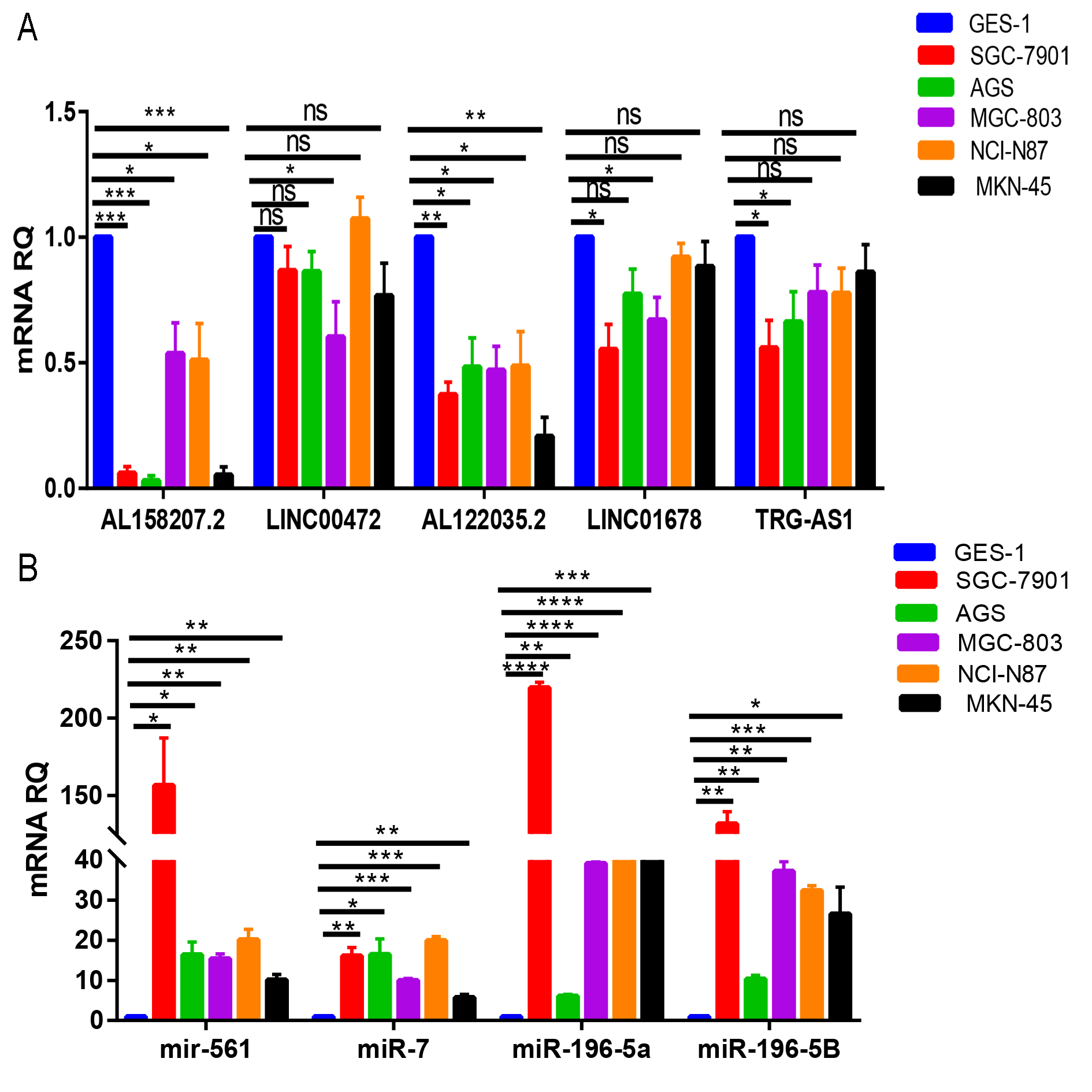

Supplement: Supplementary file 5 [file Image5.TIF]
